# Phosphatidylethanol as an outcome measure in treatment aimed at controlled drinking

**DOI:** 10.1093/alcalc/agae070

**Published:** 2024-10-04

**Authors:** Anders Hammarberg, Stina Ingesson Hammarberg, Susanna Redegren Cuellar, Joar Guterstam

**Affiliations:** Department of Clinical Neuroscience, Centre for Psychiatry Research, Karolinska Institutet, Stockholm Health Care Services, Norra Stationsgatan 69, 7th Floor, 113 64 Stockholm, Sweden; Department of Clinical Neuroscience, Centre for Psychiatry Research, Karolinska Institutet, Stockholm Health Care Services, Norra Stationsgatan 69, 7th Floor, 113 64 Stockholm, Sweden; Department of Clinical Neuroscience, Centre for Psychiatry Research, Karolinska Institutet, Stockholm Health Care Services, Norra Stationsgatan 69, 7th Floor, 113 64 Stockholm, Sweden; Department of Clinical Neuroscience, Centre for Psychiatry Research, Karolinska Institutet, Stockholm Health Care Services, Norra Stationsgatan 69, 7th Floor, 113 64 Stockholm, Sweden

**Keywords:** PEth, controlled drinking, alcohol biomarkers

## Abstract

**Background:**

Phosphatidylethanol (PEth) is a specific marker of alcohol intake, used both as a screening method for hazardous use and as an outcome measure in the treatment of alcohol use disorder (AUD). However, what cut-off values to apply for hazardous use in a treatment setting is still unclear. We aimed to investigate the correlation between PEth and self-reported drinking and identify the optimal cut-off for hazardous use, for patients with AUD and a stated goal of controlled drinking.

**Methods:**

We used data from a randomized controlled trial of two different psychological treatments aiming for controlled drinking, conducted within specialized addiction care in Stockholm, Sweden. A total of 181 patients left samples that could be included in the current analysis. Outcomes were measured at five different time points over 2 years of follow-up. PEth 16:0/18:1 values were correlated with subjective reports of recent drinking based on the Timeline Follow-Back Method.

**Results:**

The correlation between PEth and self-reported alcohol intake increased significantly over time, with the weakest correlation found at baseline (Spearman’s ρ = 0.42) and the strongest at the 104-week follow-up (ρ = 0.69). When used to indicate hazardous drinking according to Swedish guidelines (≥10 units per week), receiver operating characteristic analysis revealed PEth ≥ 0.22 μmol/l to be the optimal cut-off.

**Conclusions:**

PEth is a useful outcome measure that can be used to validate subjective reports of current drinking. In a treatment setting aimed at controlled drinking, the accuracy of patients’ self-report measures seems to improve over time. In this context, a PEth value of ≥0.22 μmol/l is a sensitive and specific indicator of hazardous drinking.

## Teaser

**Short Summary:** We characterized the correlation between phosphatidylethanol (PEth) and subjective reports of current drinking, in a treatment setting aimed at controlled drinking. We found that the accuracy of self-report measures improved over time, and that a PEth value of ≥0.22 μmol/l is a sensitive and quite specific indicator of hazardous drinking.

## Introduction

Phosphatidylethanol (PEth) is a specific marker of alcohol intake (Alling et al. 1983; Isaksson et al. 2011), which is used clinically both as a screening method for hazardous drinking and in treatment of alcohol use disorder (AUD). An objective measure of alcohol consumption such as PEth can be a valuable addition to the information obtained from clinical interviews and self-report instruments since subjective reports of alcohol intake are not always accurate due to several different reasons. Some patients may have problems with recall or difficulties in quantifying alcohol intake. There can also be psychological mechanisms that introduce bias, such as perceived stigma related to excessive drinking, uncertainty about the consequences of providing such information, or wanting to match the assumed expectations of a treatment provider. In other instances, exaggerated alcohol consumption might be reported, to emphasize one’s treatment needs. Measuring PEth can therefore be useful in a number of clinical situations, provided that it is accurately interpreted in the context of the individual case. For similar reasons, PEth has the potential to be a valid outcome measure in research for the evaluation of treatments for AUD, and its greater sensitivity and specificity make it superior to other markers such as carbohydrate-deficient transferrin (CDT) and gamma-glutamyl transferase (GGT) (Walther et al. 2015).

A challenge in using PEth in clinical practice has been correlating a certain PEth concentration to a specific amount and duration of alcohol consumption (Helander et al. 2019b). Cut-offs for different levels of alcohol consumption have been suggested. In Sweden, PEth levels above 0.30 μmol/l are routinely considered to be indicative of hazardous use, whereas levels between 0.05 and 0.30 μmol/l indicate moderate drinking. Levels <0.05 μmol/l indicate none, or low and sporadic, alcohol consumption (Helander and Hansson 2013). However, it is still debated whether the cut-off of 0.30 μmol/l is optimal for identifying hazardous drinking (Hahn et al. 2016; Skråstad et al. 2023). To our knowledge, there has not been any specific studies of this cut-off in relation to the former Swedish definition of hazardous drinking as >9/14 standard drinks per week in women/men, nor in relation to the current definition of ≥10 standard drinks (12 g of ethanol) per week, in both men and women (this definition was updated in 2023).

However, a number of studies have investigated the relationship between PEth values and self-reported alcohol intake, in various samples including patients, healthy volunteers, and the general population (Walther et al. 2015; Schröck et al. 2017; Helander et al. 2019b; Skråstad et al. 2023). For instance, Helander et al. included 36 individuals with AUD in an outpatient treatment setting and found that the PEth threshold of ≥0.30 μmol/l corresponded to a reported average daily intake of ~60 g ethanol (Helander et al. 2019b). In an observational study of 300 healthy volunteers, Schröck et al. found 74 ‘excessive drinkers’, with an average daily intake of 12.5 g ethanol, to have a mean PEth value of 0.16 μmol/l (Schröck et al. 2017). In a randomized experiment, Kechagias et al. included 44 healthy volunteers who were instructed to drink 16 g (women) or 32 g (men) of ethanol/day over 3 months (Kechagias et al. 2015). The mean PEth concentration after this period was only 0.022 μmol/l. However, there was a substantial variability in the observed PEth levels, which might have to do with a lack of adherence to the instructions on alcohol intake.

Based on the results of their large population study, Skråstad et al. argued that a PEth value as low as 0.08 μmol/l would be a suitable cut-off for the identification of individuals drinking >2 alcohol units per day (i.e. > 24 g of ethanol), which is currently considered ‘hazardous drinking’ in many countries (Skråstad et al. 2023). However, their analyses were based on questionnaires asking about alcohol habits in general, rather than specifically mapping the days and weeks before the PEth measurements, which is possible to accomplish with the Timeline-Follow Back (TLFB) method (Sobell et al. 1986). It is therefore unclear if such a low cut-off is reasonable as a marker of current hazardous drinking in a treatment setting. The 2022 Consensus of Basel document on the use of PEth, instead suggested that 200 ng/ml, corresponding to 0.28 μmol/l, is a suitable cut-off for “excessive alcohol consumption” (Luginbühl et al. 2022).

Another question is whether the accuracy of self-reported drinking measures changes over time, in patients treated for AUD. This would of course lead to a change in the correlation between self-report and PEth values. One might expect an improvement in the precision of the self-reports as a result of engaging in treatment. Commonly, patients experience an increased awareness of their drinking behaviors and also work specifically with monitoring their alcohol consumption by the use of an ‘alcohol diary’ in many behavioral treatments for AUD, including behavioral self-control training (BSCT)(Hester and Miller 2003; Jemberie et al. 2023; Jirwe et al. 2024). Furthermore, if the therapeutic relationship between patient and clinician improves over time, it may be expected that patients will report their alcohol consumption more accurately. On the other hand, the ongoing relationship with a treatment provider may introduce other biases that could lead to inaccurate reporting, e.g. a desire to report a positive treatment outcome. Hypothetically, such problems might be more pronounced in a strictly abstinence-oriented treatment setting, where any alcohol intake could be perceived as a ‘failure’ for both the patient and the therapist. The desirability bias in reporting to the therapist may be less strong in treatments aiming for controlled drinking, where alcohol use is expected.

Because of the increased use of PEth in both research and clinical practice, there is a need for more precise estimates of the relation between specific PEth values and recent alcohol consumption. This is particularly relevant in treatment aiming at controlled drinking, in which is not self-evident what PEth values may constitute a positive outcome. From a clinical perspective, it might also be relevant to know whether the validity of self-report measures of alcohol use changes throughout treatment.

We therefore aimed to investigate the correlation between PEth values and recent alcohol intake (last 14 days), as reported with the TLFB method, and whether the correlation between PEth and TLFB changes during treatment, from the first measurement to the last follow-up. We also studied the correlation between PEth values and other markers commonly used for identifying hazardous alcohol use (CDT and GGT). Finally, we aimed to analyze what cut-off level would be suitable, when using PEth as an outcome measure for hazardous drinking.

While some of these questions have been explored in previous studies, most of the existing experimental and clinical studies suffer from small sample sizes, and the larger, population-based studies lack the temporal precision needed to answer these research questions (Helander et al. 2019b; Skråstad et al. 2023). In this study, we used data from a recently conducted clinical trial with individuals fulfilling the criteria for AUD (Hammarberg et al. 2024). The patients (*n* = 181) did not have a goal of abstinence, which resulted in significant variability in PEth values throughout the trial.

## Materials and Methods

We used data from a randomized clinical trial, including 250 individuals with AUD conducted in Stockholm, Sweden from August 2017 to December 2022. The trial aimed to investigate whether BSCT was superior to Motivational Enhancement Therapy (MET) in reducing alcohol consumption and related consequences. The primary outcome of the main study was the difference in weekly alcohol consumption between groups at 26 weeks. Patients were assessed at baseline, 12, 26, 52, and 104 weeks. The study was conducted at three addiction outpatient clinics at Stockholm Centre for Dependency Disorders, Sweden. The study was approved by the Regional Ethics Board in Stockholm (DNR: 2016/1113–312) and registered at ISRTCN: 14539251. For full details of the main clinical trial, see Ingesson-Hammarberg et al. 2024 (Hammarberg et al. 2024).

### Participants and procedure

The study sample comprised patients newly admitted to the clinics either through self-referrals or via advertisements. During their initial assessment meeting at the clinic, patients were invited to participate in the trial comparing two treatments for controlled drinking. If willing to participate, patients underwent a standardized screening procedure with a trained assessor at the study site. Eligible participants were adult individuals aged between 18 and 70 years, diagnosed with AUD according to the DSM-5, and with an expressed treatment goal of controlled drinking. Exclusion criteria included the presence of any other substance use disorder except nicotine, severe psychiatric conditions (such as suicidal ideation, severe major depression, untreated bipolar disorder, or psychotic disorder), recent initiation of antidepressant medication (<30 days), any inpatient AUD treatment- or alcohol intoxication the last 12 months, or significantly elevated liver enzymes (three times the clinical cutoff). If fulfilling the eligibility criteria and consenting to participate, patients were included and thereafter randomized to one of the treatments.

### Measures

Baseline measures of clinical characteristics and sociodemographic data were collected from all patients as part of the trial protocol. Patients were diagnosed with AUD and screened for other psychiatric diagnoses using the Mini International Neuropsychiatric Interview (MINI) (Sheehan et al. 1998). Follow-up assessments were made after 12, 26, 52, and 104 weeks.

Blood tests including PEth, CDT, and GGT were taken at baseline and every follow-up. PEth 16:0/18:1, being the most prevalent homologue and a good proxy for total PEth, was analyzed using LS-MS/MS (Helander and Hansson 2013). The results were reported in μmol/l and values <0.05 were reported as negative, in accordance with national standards in Sweden (Helander and Hansson 2013). All values <0.05 were therefore set as 0 in our analyses.

Alcohol consumption was measured according to the TLFB method, including the 90 days preceding the day of assessment. The TLFB method tracks daily alcohol intake retrospectively, measured in standard drinks (in this study equivalent to Swedish standards of 12 g of pure ethanol). In the current study, the total self-reported consumption of the 14 days preceding the date of the blood test was included to analyze the correlation between self-reported consumption and PEth values. This choice of the period of days included in the analysis was based on the results from Helander et al. (Helander et al. 2019b), demonstrating that PEth most accurately measures alcohol consumed in the preceding 14 days, a finding that was replicated in this dataset (Supplementary Fig. 1). The Alcohol Use Disorders Identification Test (AUDIT) total score was used as a measure of the severity of alcohol problems (Saunders et al. 1993).

### Interventions

Patients in the trial received one of the two treatments; BSCT or MET. BSCT is a manual-based cognitive-behavioral treatment (Miller and Muñoz 2013; Hammarberg and Wallhed 2015) comprising five sessions. The treatment includes self-monitoring of alcohol consumption, identification of risk situations, and strategies for increasing rate control, and relapse prevention. The other treatment was MET, which is a manual-based treatment consisting of four sessions. The manual used was the Swedish MET manual, adjusted for being used in Swedish treatment settings (Hammarberg *et al.* 2021). The manual includes an initial assessment and a personalized feedback session. The three subsequent sessions are conducted in line with the principles of Motivational Interviewing (Miller 1994). For more detailed information on these interventions, please see the published manuscript on the trial’s primary outcome (Hammarberg et al. 2024).

### Data preparation and statistical analyses

All available data were used and were not split up between treatment groups since the questions of this study were not related to treatment allocation. Correlations were quantified using Spearman rank correlation, because of outliers in the data. As the chosen analyses were deemed to be robust against outliers, these were kept in the dataset. To examine the ability of PEth to identify hazardous drinking, receiver operating characteristic (ROC) curves were created, describing the relation between sensitivity and specificity. The optimal cut-off value was identified with Youden’s J statistic, which calculates the cut-off where the sum of sensitivity and specificity is maximized. Hazardous drinking was defined as average weekly number of drinks larger than or equal to 10 for the last 2 weeks. All analyses were performed with R version 4.3.1 (R Core Team 2021).

## Results

Demographics and baseline clinical characteristics of the patient sample are detailed in Table 1. Briefly, the patients had a moderate or severe AUD, were on average slightly above 50 years of age, and about half of them were female.

**Table 1:** Baseline measures for the patients who contributed data to this study (*n* = 181)

**Variable**	**Measure**	
Gender		
Male	*n* (%)	87 (48.1%)
Female	*n* (%)	94 (51.9%)
Age	Median (range)	53 (28–70)
PEth	Median (range)	0.4 (0–2.9)
CDT	Median (range)	1.7 (0.2–9.8)
GGT	Median (range)	0.5 (.1–4.3)
Average number of standard drinks per week	Median (range)	21.2 (0–77.4)
AUDIT score	Median (range)	19 (6–35)
Number of DSM criteria for alcohol use disorder	Median (range)	5.5 (2–11)

Note: PEth = phosphatidylethanol (μmol/l); CDT = carbohydrate deficient transferrin (%); GGT = gamma-glutamyltransferase (microkat/l).

A total of 250 patients were included in the clinical trial, but because of dropouts, missing PEth samples, or TLFB reports, the number of patients with complete data at each time point was 181 at baseline and declined to 100 at the 104-week follow-up. A number of patients had to be excluded because the blood sampling had been delayed for a few days due to practical reasons, which meant that the PEth values could not be matched directly to TLFB data of the 14 days preceding the sample. The 181 patients included in the final sample had a higher proportion of women but did not differ in age, compared to the 69 patients who could not be included in these analyses. Most patients reduced their consumption and maintained this change in drinking throughout the trial (Hammarberg et al. 2024; Ingesson-Hammarberg et al. 2024) (Supplementary Fig. 2).

**Figure 1:**
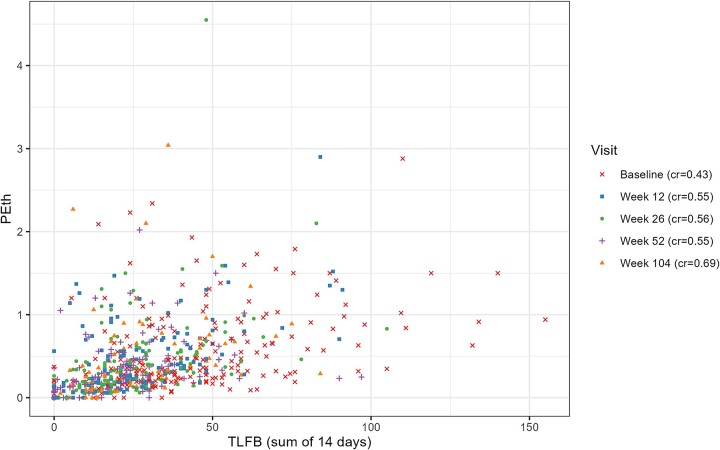
PEth and self-reported drinking, from baseline (week 0) to last follow-up (week 104). PEth values are reported in μmol/l and drinking is quantified as the reported total number of standard drinks (12 g of ethanol) consumed during the 14 days preceding the day of the PEth sample. cr: correlation between PEth and TLFB (Spearman’s ρ)

Figure 1 depicts the relationship between PEth values and the number of standard drinks consumed in the last 14 days for all time-points of measurement, as reported with the TLFB method.

**Figure 2:**
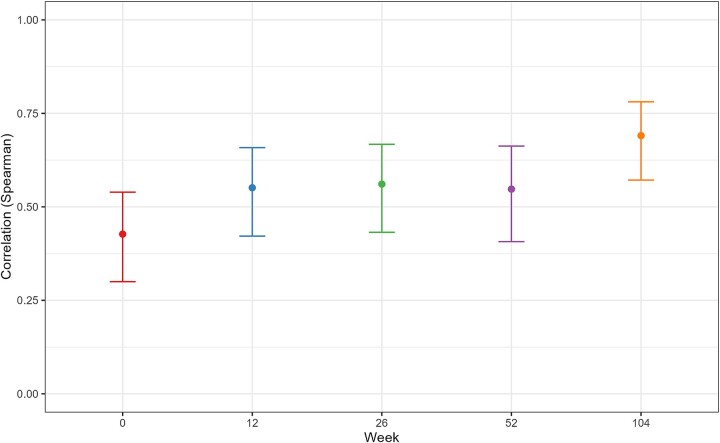
Correlations between PEth and self-reported drinking in the last 14 days. Correlation values are Spearman’s ρ, with 95% confidence intervals.

At baseline, the correlation between PEth values and self-reported drinking was relatively weak, with a Spearman’s ρ of 0.42. However, the correlation became stronger with time, reaching a level of ρ = 0.69 at the 104-week follow-up, a statistically significant increase in strength of correlation from baseline (Fig. 2).

**Figure 3:**
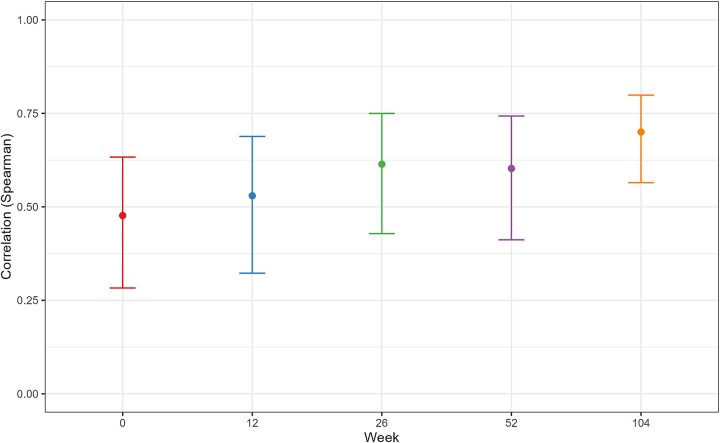
Correlations between PEth values and self-reported drinking in the last 14 days, including only subjects with complete data at both baseline and week 104 (*n* = 77).

A possible explanation for the increased correlation could be a selection effect, meaning that patients with more accurate self-reports at baseline had a higher tendency to stay in the study through long-term follow-up compared to patients with less accurate self-reports. Examining only subjects who provided complete data both at baseline and at the 104-week follow-up, it appears that this is not the case. Their correlation values at baseline closely resembled those of the whole sample, and the temporal trend was similar (Fig. 3).

**Figure 4:**
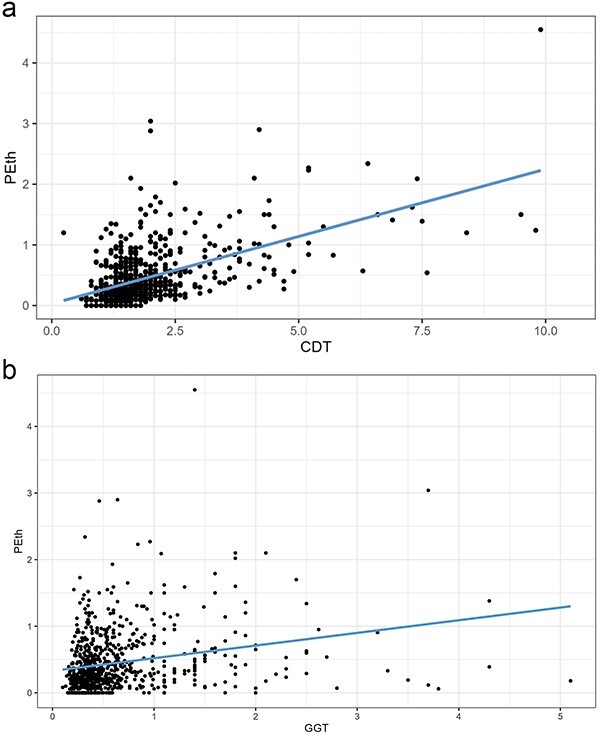
(a) Correlation between PEth and CDT. (b) Correlation between PEth and GGT.

When comparing PEth with other biomarkers, we found a moderately strong correlation with CDT (Spearman’s ρ = 0.54) and a weak correlation with GGT (0.24) (Fig. 4a and b).

**Figure 5:**
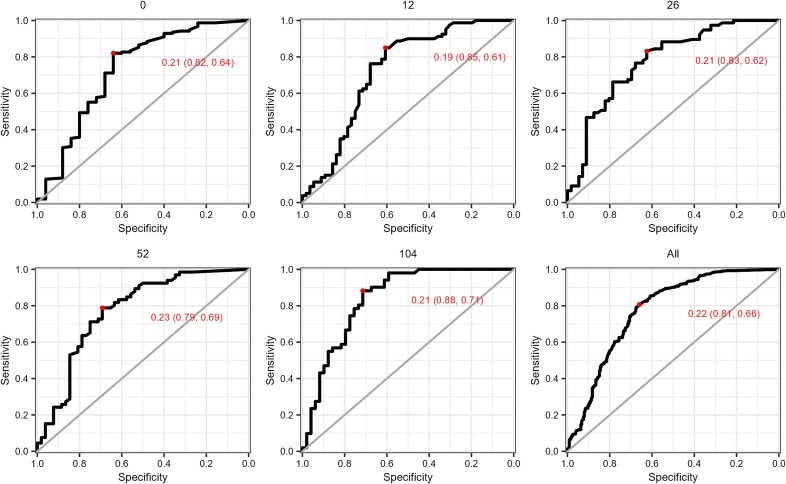
ROC analyses estimating the PEth value corresponding to the cut-off for hazardous drinking (≥10 standard drinks per week).

In the current trial, the patients’ stated goal was controlled drinking. A common goal among patients is to attain drinking levels below what is defined as hazardous drinking, which varies between countries. In Sweden, current guidelines define hazardous drinking as a weekly alcohol intake of 10 or more standard drinks. When estimating a PEth value corresponding to this drinking level, ROC analysis revealed ≥0.22 μmol/l to be the optimal cut-off (Fig. 5). Using data from the 104-week follow-up, which had the best correlations between PEth and self-reported drinking, this cut-off exhibited good sensitivity (0.88) and moderately good specificity (0.71).

## Discussion

In this analysis of data from a clinical trial of AUD treatment aiming at controlled drinking, we found that PEth correlated moderately with self-reports of alcohol intake. This correlation became stronger over time, from baseline to end of follow-up after 2 years. These findings suggest that the validity of self-report measures improves with time, possibly as a result of increased awareness and attention to drinking behaviors, and of decreased alcohol intake.

A strength of PEth as an alcohol marker is its absolute specificity, where abstinence always corresponds to a PEth value of 0. However, positive PEth values are more challenging to translate into specific drinking levels. In the current study, the correlations between PEth and self-reported drinking are in the same range as in a number of earlier clinical studies (Isaksson et al. 2011; Kechagias et al. 2015; Walther et al. 2015; Schröck et al. 2017; Helander et al. 2019b). Although the correlations became stronger with time, the inter-individual variabilities in PEth formation and half-life of the PEth response (Hill-Kapturczak et al. 2018; Helander *et al.* 2019a) imply that there cannot be a perfect correlation, even with 100% accurate self-report measures. Indeed, earlier studies have shown that there can be significant differences both in the formation and elimination of PEth, varying up to 3-fold between different persons. The reasons for this are incompletely understood, but could involve genetic or acquired differences in relevant enzymes, such as phospholipase D (Hill-Kapturczak et al. 2018). This makes it less straightforward to use PEth as an outcome measure when the treatment goal is controlled drinking, rather than complete abstinence.

Therefore, we also aimed to identify the PEth cut-off corresponding to the threshold for hazardous drinking, which in Sweden is currently set at 10 units per week for both men and women. We found ≥0.22 μmol/l to be the optimal cut-off for identifying drinking levels of ≥10 units per week, during the 2 weeks prior to the PEth sampling. This cut-off demonstrated both a high degree of sensitivity and specificity. In situations where minimizing the risk of false positives is warranted, the cut-off could be slightly higher. The current Swedish standard of labeling PEth values ≥0.30 μmol/l as ‘indicating high alcohol consumption’ is an example of this. With such a high cut-off, there will be quite a few false negatives scoring just below 0.30, but a low risk of any false positives. The choice of cut-off may therefore depend on the specific purpose of the testing.

The PEth values corresponding to a weekly intake of ≥10 units according to our study (≥0.22 μmol/l) are significantly higher than those reported by Skråstad et al. to indicate a daily intake of >14 units per week (≥0.08 μmol/l) (Skråstad et al. 2023). This may be due to methodological differences. Skråstad used a population sample and correlated PEth values with data from questionnaires about general alcohol habits, while our study included patients in treatment for AUD, who provided structured self-report data on their drinking specifically on the days preceding the PEth measurement. Of course, the latter approach gives a far better temporal resolution, and perhaps also a more accurate self-report. Our results are also compatible with earlier data from studies with healthy volunteers and alcohol dependent patients, even if differences in methods and reporting make them somewhat challenging to compare directly (Walther et al. 2015; Schröck et al. 2017; Helander et al. 2019b). We therefore argue that a cut-off of 0.22 μmol/l, or somewhat higher if specificity should be prioritized, is more suitable to use as a follow-up measure in a treatment setting. In population-based screening, a single PEth measurement is not a sensitive marker of past-year hazardous drinking, since the application of a very strict cut-off, allowing for the detection of alcohol intake during the last couple of weeks, would have low specificity for hazardous drinking (Schröck et al. 2017).

### Strengths and limitations

This study included a relatively large sample of participants, who were in treatment for AUD and who exhibited a range of positive PEth values. We used precise data on the timing of the blood sample measurements in relation to self-reported alcohol consumption. The repeated measurements enabled us to study how the relationship between PEth and self-reported consumption changed over an extended period. However, there are also some limitations to consider. For instance, we did not have data on participants’ body mass index, a factor that may affect PEth sensitivity (Hahn et al. 2021). Another limitation of this and many other studies investigating the association between PEth and alcohol consumption is the lack of precise, objective measurements of alcohol intake, although such measures (e.g. direct observations of patients drinking) would present practical and ethical challenges. So far, experimental studies performed in a monitored setting have typically been restricted to single drinking sessions and subsequent PEth measurements. In a somewhat longer study, Gnann et al. instructed their healthy participants to drink 5–9 units daily, in monitored sessions for 5 days in a row, which led to PEth values ranging from 0.11 to 0.77 μmol/l (Gnann et al. 2012). However, 5 days is of course also a relatively short period of time, and the results are therefore hard to translate into the clinical situation of patients with AUD.

Relying on retrospective self-report entails a risk of bias, including difficulties in remembering past consumption (recall bias), which might be an even more prominent issue for individuals with high drinking levels. The patients might also be unaccustomed to estimate their precise alcohol consumption. Moreover, social desirability may cause patients to self-report a lower alcohol consumption, in order not to disappoint the therapist or other health care professionals. On the other hand, all patients in this study were self-referred for their treatment, suggesting they had little reason not to give a reliable report. In addition, the treatment goal of the trial was controlled drinking, which probably reduced any potential pressure to report complete abstinence, making a conscious underreporting of alcohol consumption less likely.

Another potential source of bias is the risk of post-sampling PEth formation that might occur if a patient has a high blood alcohol concentration when leaving his or her blood sample. Although this is probably not any major issue in our study, future research may employ other sampling techniques that minimize this risk, such as dried blood spots with a phospholipase D inhibitor (Beck et al. 2021). One may also consider adding other specific alcohol markers, such as ethyl glucuronide hair testing, in order to validate the PEth data (Biondi et al. 2019). Finally, our dataset did now allow for a stringent evaluation of whether different drinking patterns (e.g. binge drinking on 1–2 days per week vs. regular daily) may have a different impact on PEth measures, despite a similar number of total drinks per week. The problem is that self-report may become less reliable in a binge situation, and the time lapse from end of binge drinking to the PEth measurement introduces a lot of variability. Specific, experimental study designs may be needed to resolve this question.

In conclusion, this study adds to the literature on the clinical and scientific use of PEth and suggests that values ≥0.22 μmol/l indicate hazardous drinking according to Swedish guidelines, i.e. a current weekly alcohol intake of ≥10 units. With repeated measures, self-reported alcohol intake correlates more strongly with PEth values over time, indicating an improvement in the precision of such reports. With more specific cutoffs that are indicative of low-risk drinking, PEth is a valuable complement to self-report measures in the assessment and follow-up of patients with AUD. It should therefore be included as an objective outcome measure in treatment studies for both abstinence and controlled drinking outcomes.

## Supplementary Material

Supplementary_material_v2_agae070

## Data Availability

The data that support the findings of this study are available from the corresponding author upon reasonable request.
