# Supplementary material for: Phosphatidylethanol as an outcome measure in treatment aimed at controlled drinking
Source: Alcohol Alcohol. 2024 Oct 4;59(6):agae070. doi: 10.1093/alcalc/agae070 (PMC11452308; doi:10.1093/alcalc/agae070)
Supplement: Supplementary_material_v2_agae070 [file supplementary_material_v2_agae070.docx]

## **Supplementary material**


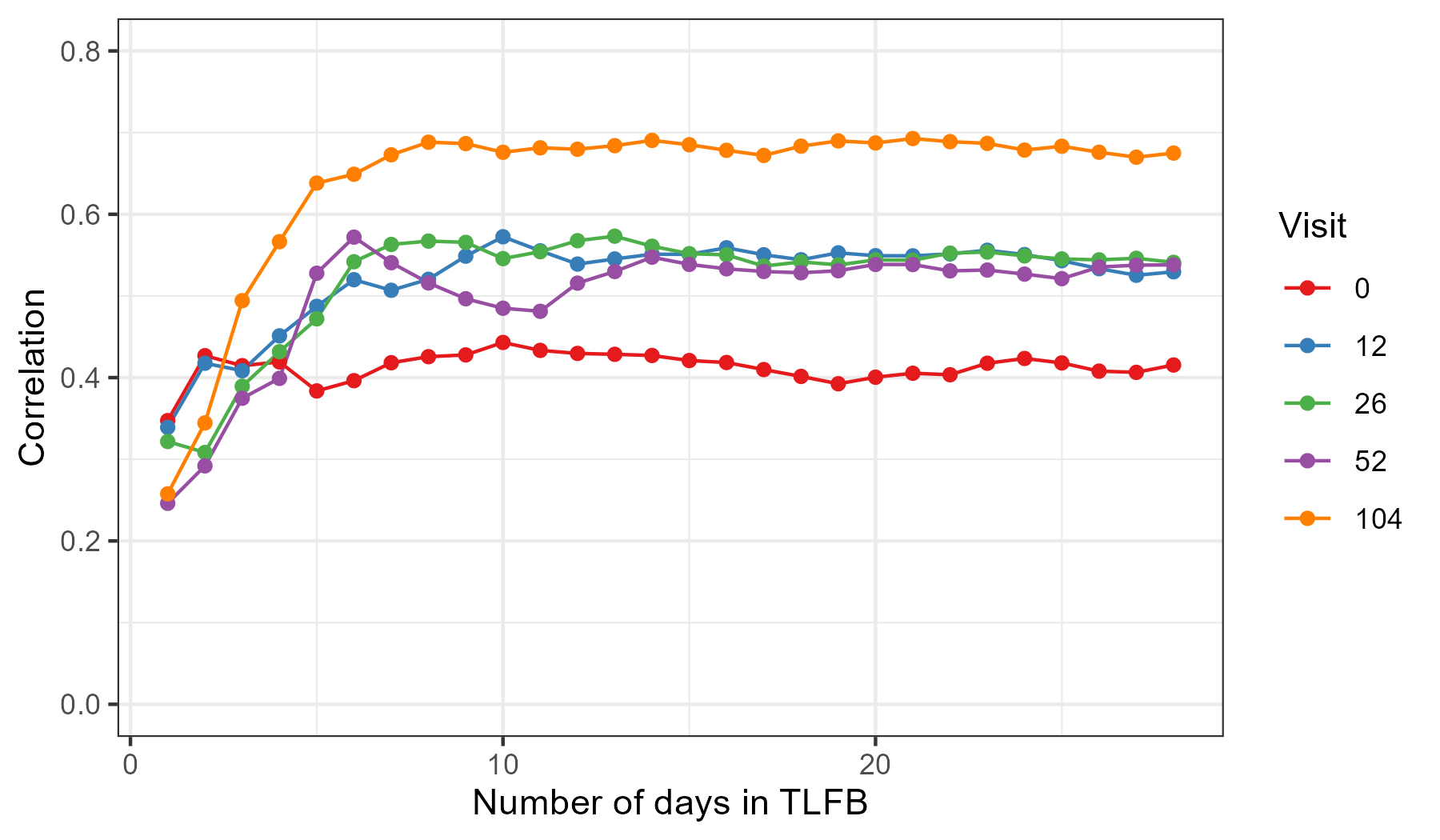


Figure S1: Correlation between PEth values and self-reported number of standard drinks consumed in the preceding days. The y axis describes Spearman’s ρ, and the x axis the number of days included in the calculation.


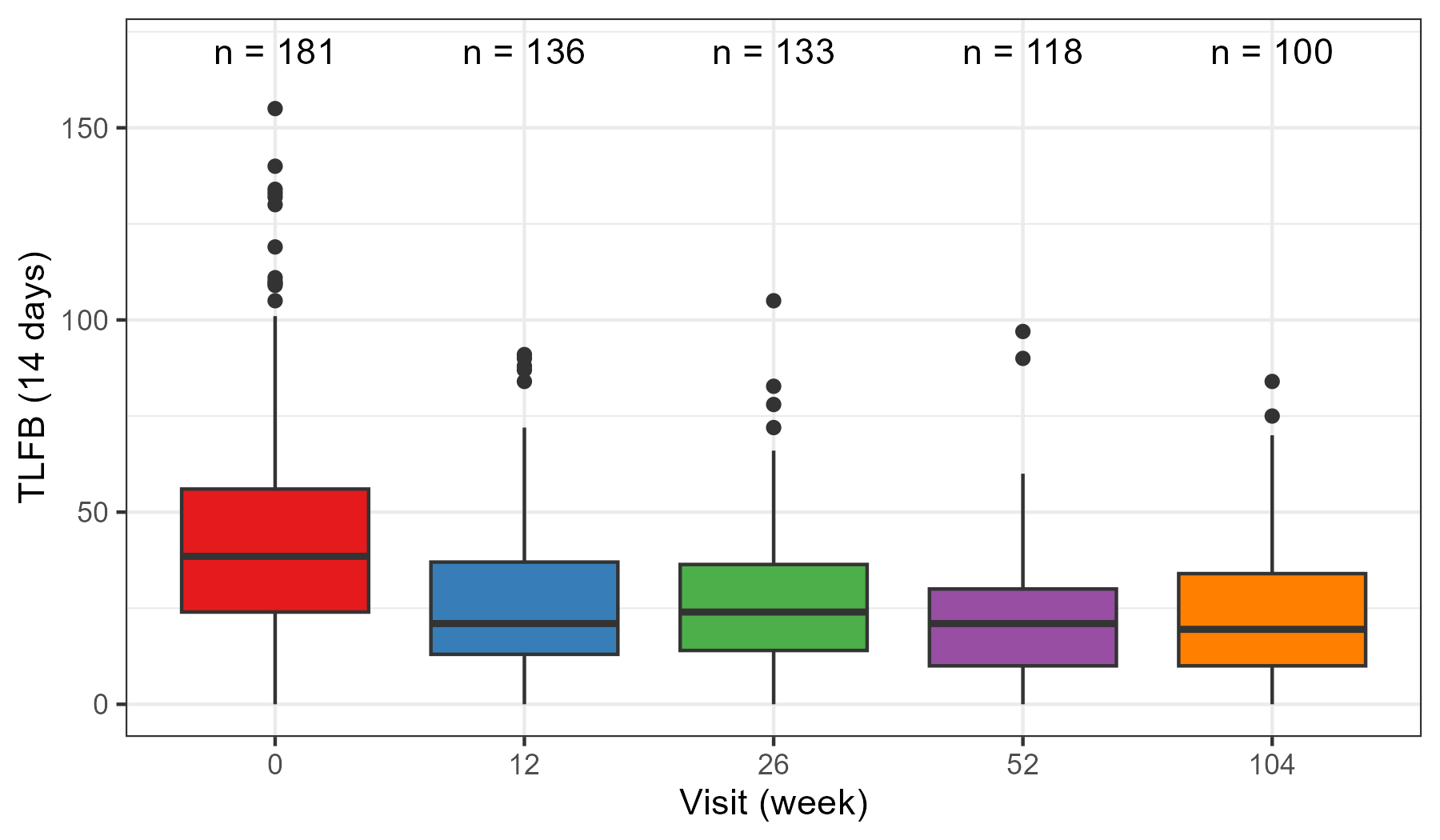


Figure S2: Box plot depicting drinking levels (number of standard drinks per 14 days) at the different time points of follow-up.
